# Association between body roundness index and metabolic syndrome in middle-aged and older adults: a prospective cohort study in China

**DOI:** 10.3389/fpubh.2025.1604593

**Published:** 2025-07-09

**Authors:** Haiwei Li, Jing Zhang, Huifang Wang, Liang Luo

**Affiliations:** School of Physical Education, Shanxi Normal University, Taiyuan, China

**Keywords:** body roundness index, metabolic syndrome, CHARLS, middle-aged and older adults, China

## Abstract

**Background:**

The Body Roundness Index (BRI), an emerging anthropometric parameter calculated from height and waist circumference ratios, currently lacks substantive evidence delineating its etiological connections with metabolic syndrome (MetS) development. Its predictive utility for MetS and clinical applicability remain poorly understood. This study aimed to investigate the association between BRI and the risk of MetS in middle-aged and older adults in China, using both cross-sectional and prospective cohort analyses. We hypothesized that higher BRI is associated with an increased risk of MetS.

**Methods:**

The cross-sectional analysis utilized data from the China Health and Retirement Longitudinal Study (CHARLS), comprising 9,398 participants, while the longitudinal analysis was based on a prospective cohort of 5,934 individuals from the same study, followed over a 4-year period. The BRI was calculated using height and waist circumference. Logistic regression and Cox proportional hazards regression models were employed to evaluate associations between BRI and MetS. To ensure the robustness of the findings, restricted cubic spline plots, subgroup analyses, and sensitivity analyses were conducted.

**Results:**

After adjusting for covariates (including age, gender, education, smoking status, drinking status, etc.), cross-sectional analyses revealed that participants in the medium BRI tertile (OR = 4.99, 95% CI: 3.07–8.11) and the high BRI tertile (OR = 13.66, 95% CI: 8.57–21.79) had a significantly higher risk of MetS compared to the low BRI reference group (*p* < 0.001). Longitudinal analyses demonstrated that the medium BRI group had a 2.71-fold increased risk of MetS (HR = 2.71, 95% CI: 2.29–3.21, *p* < 0.001), while the high BRI group exhibited a 4.64-fold increased risk (HR = 4.64, 95% CI: 3.94–5.47, *p* < 0.001) relative to the low BRI group. Restricted cubic spline analyses indicated a nonlinear dose–response relationship between BRI and MetS risk (P for nonlinearity < 0.001).

**Conclusion:**

Elevated BRI is significantly associated with an increased risk of MetS in middle-aged and older adults. Therefore, prospective cohort studies employing longitudinal designs and intervention assessments are needed to determine whether BRI can serve as a modifiable risk marker for MetS.

## Introduction

Metabolic syndrome (MetS), a prevalent metabolic disorder characterized by elevated blood pressure, dyslipidemia [increased triglycerides (TG) and reduced high-density lipoprotein cholesterol (HDL-C)], impaired fasting glucose and central obesity (1), has shown a sustained upward trend among the adult population in China, as evidenced by multiple epidemiological studies (2–5). A 2020 nationwide study in China reported that 33.38% of adults met the diagnostic criteria for MetS (6). The rising prevalence of this condition has garnered increasing scholarly attention in recent years (7). Multiple studies demonstrate an association between gut microbiota diversity and metabolic disorders (8, 9). Moreover, MetS is strongly associated with a higher risk of chronic comorbidities, including malignancies, neurological disorders, and non-alcoholic fatty liver disease. These conditions are linked to systemic dysregulation in reproductive, lipid, and circulatory homeostasis, as well as elevated all-cause mortality (10–15).

The pathogenesis of metabolic syndrome (MetS) is closely linked to obesity (1, 16, 17), with abdominal adiposity serving as a critical pathophysiological determinant (18). Conventional anthropometric indices for assessing obesity—such as body mass index (BMI), waist circumference (WC), and waist-to-hip ratio (WHR)—have demonstrated significant associations with MetS risk in epidemiological studies (19–21). However, these traditional metrics exhibit notable limitations in predicting the onset of MetS. For instance, body mass index (BMI) demonstrates limited capacity to differentiate between lean muscle mass and adipose tissue composition, while its measurement accuracy may be compromised by confounding variables (22, 23). Multiple studies have reported significant variability in the predictive capacity of these indicators for MetS, with inconsistencies observed across different research findings (24, 25).

To address the limitations of conventional anthropometric measures, Thomas et al. introduced the Body Roundness Index (BRI) in 2013, an obesity indicator that integrates waist circumference and height (26, 27). BRI demonstrates enhanced discriminative validity in quantifying adipose tissue compartmentalization, particularly in differentiating visceral adiposity from subcutaneous fat reservoirs (22). Emerging evidence suggests that BRI outperforms traditional body composition indicators in predicting risks for diverse clinical endpoints, including MetS, cardiometabolic disorders, and renal pathologies (27–29). Notably, prior studies have established a dose–response relationship between incremental increases in BRI and heightened susceptibility to MetS (27, 30). However, the scientific literature currently lacks methodologically rigorous longitudinal prospective cohort studies that comprehensively investigate the association between BRI and MetS in Chinese populations, representing a critical evidence gap in population-specific metabolic research. Moreover, it remains unclear whether BRI predicts MetS risk independently of other obesity measures in middle-aged and older Chinese adults. This study utilizes data from the China Health and Retirement Longitudinal Study (CHARLS) spanning 2011 to 2015 to examine both cross-sectional and longitudinal associations between BRI and MetS risk among middle-aged and older Chinese adults. Furthermore, the study highlights the potential of BRI as a novel predictor of MetS and provides an evidence-based framework for targeted preventive strategies.

## Methods

### Study population

The CHARLS is a nationally representative cohort of community-dwelling Chinese adults aged 45 years and older (31). Initiated in 2011 as a baseline survey, the study recruited 17,705 participants from 150 counties across 28 provinces in China. Follow-up assessments were conducted every 2 to 3 years (31). The CHARLS protocol adhered to the principles of the Declaration of Helsinki and was approved by the Biomedical Ethics Committee of Peking University (IRB00001052-11015 and IRB00001052-11014). Written informed consent was obtained from all participants prior to their enrollment.

**Figure 1:**
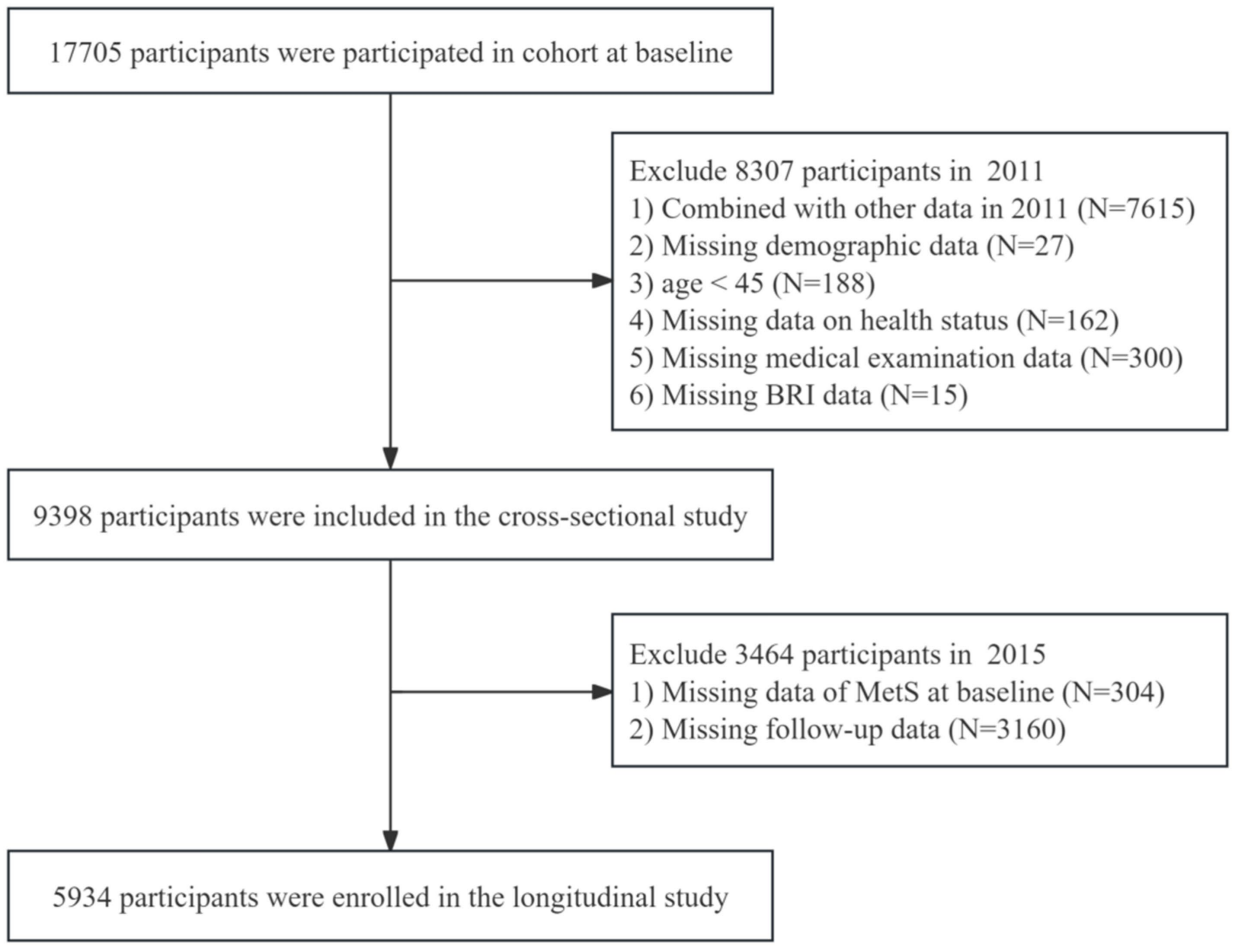
Participant selection flowchart.

This study utilized data from three CHARLS survey waves (2011, 2013, and 2015), integrating cross-sectional and longitudinal designs to rigorously examine the association between BRI and MetS. Anthropometric measurements including height, weight, and waist circumference were obtained during the 2011 baseline assessment, with blood samples collected in 2011 and 2015. The inclusion criteria were as follows: (1) Participants aged 45 years and older at baseline (2011); (2) Complete BRI data at baseline. The exclusion criteria included: (1) Prevalent MetS cases at baseline (2011); (2) Missing MetS status data in the 2015 follow-up; (3) Incomplete longitudinal data during follow-up. The final analytical cohort comprised 5,954 participants for regression analyses evaluating the predictive utility of baseline BRI for incident MetS. Figure 1 shows the participant selection flowchart.

### Assessment of body roundness index

The BRI was calculated using the following formula: $\mathrm{BRI}=364.2-365.5\times \sqrt{(1-{\left(\mathrm{WC}÷2\pi \right)}^{2}÷{\left(0.5\times H\text{eight}\right)}^{2}}.$ Waist circumference was measured using a non-elastic tape measure to make a horizontal circle around the waist at the position of the belly button, and standing height was assessed with a stadiometer. Both measurements were recorded to the nearest 0.1 cm (26, 32). Due to the lack of standardized classification criteria, BRI values were categorized into tertiles for analysis. Participants were stratified into three groups based on BRI percentiles: <33rd percentile, 33rd to <67th percentile, and ≥67th percentile (33).

### Assessment of MetS

MetS cases were identified using the diagnostic criteria established by the National Cholesterol Education Program Adult Treatment Panel III (NCEP ATP III, 2005) (34, 35), with modifications based on Chinese-adjusted definitions (36, 37). The syndrome was defined by the presence of three or more of the following five components: (1) elevated waist circumference for central obesity: ≥80 cm for females or ≥90 cm for males; (2) elevated triglyceride (TG) levels: ≥150 mg/dL or current use of lipid-lowering treatment; (3) reduced high-density lipoprotein cholesterol (HDL-C): <40 mg/dL for males or <50 mg/dL for females, or current use of lipid-modifying therapy; (4) elevated blood pressure: systolic blood pressure (SBP) ≥ 130 mmHg or diastolic blood pressure (DBP) ≥ 85 mmHg, or a documented diagnosis of hypertension with antihypertensive treatment; (5) elevated fasting blood glucose (FBG): FBG ≥ 100 mg/dL or a self-reported diagnosis of diabetes mellitus. Individuals meeting three or more of these criteria were classified as having MetS.

### Covariates

Trained interviewers collected demographic information through structured questionnaires. To address potential confounding factors, this study adjusted for sociodemographic, lifestyle, and health-related characteristics at baseline. These included: age (years), gender (male or female), hukou status (agricultural or non-agricultural), marital status (married or other), education level (illiterate, primary school or below, secondary school, college or above), current smoking status (yes or no), current alcohol consumption (yes or no), sleep duration (hours/day), participation in social activities (yes or no), BMI, socioeconomic status (SES), and self-reported history of chronic conditions, including hypertension, diabetes, dyslipidemia, cancer, chronic pulmonary disease, liver disease, cardiovascular disease, stroke, kidney disease, gastrointestinal disorders, psychiatric conditions, memory-related disorders (Alzheimer’s disease, brain atrophy, Parkinson’s disease), arthritis/rheumatism and asthma. The number of chronic conditions was categorized as 0, 1, or ≥ 2. BMI was calculated using the formula: $\mathrm{BMI}=W\text{eight}\;(\mathrm{kg})÷H{\text{eight}}^{2}\;(m^{2})$ . In addition, SES was categorized into low, medium and high levels.

### Statistical analysis

Continuous variables with normal distribution are expressed as mean ± standard deviation (SD); non-normally distributed variables are reported as median (interquartile range, IQR). Categorical variables were reported as frequencies with percentages. Between-group differences for continuous variables were assessed using analysis of variance (ANOVA) or Kruskal Wallis test, and Chi-square tests were used to compare categorical variables. Normality tests indicated that age and sleep duration were not normally distributed, with the exception of BMI. The results of the normality test were shown in the Supplementary Table 1.

To investigate the association between BRI and MetS, two regression models were employed. Logistic regression models were used to assess the cross-sectional association between BRI and MetS prevalence, with odds ratios (ORs) and corresponding 95% confidence intervals (CIs). For the longitudinal analysis, Cox proportional hazards regression models were applied to estimate hazard ratios (HRs) and 95% CIs for the association between baseline BRI and incident MetS during follow-up. Three sequential models were constructed: Model 1: Unadjusted for covariates; Model 2: Adjusted for age, gender, hukou status, marital status and education level; Model 3: Further adjusted for current smoking status, current alcohol consumption, sleep duration, participation in social activities, BMI, SES and chronic disease burden, based on Model 2. Additionally, restricted cubic splines (RCS) with four knots were incorporated into the fully adjusted Cox regression model to evaluate potential non-linear dose–response relationships between BRI and MetS risk. Missing values meeting the predefined exclusion criteria were excluded from the analysis. Multivariate Cox proportional hazards regression models were employed to conduct subgroup analyses stratified by age (<60 years and ≥60 years), gender, hukou status, marital status, educational level, current smoking status, current alcohol consumption, BMI (< 24 kg/m^2^ and ≥ 24 kg/m^2^), the number of chronic conditions and SES to evaluate the association between BRI and MetS risk. Sensitivity analyses were subsequently performed to assess the robustness of results through two approaches: (1) redefining MetS using exclusively laboratory blood test parameters and physical examination data, and (2) excluding participants with baseline BRI values falling within the extreme 15% range. All statistical analyses and data processing were performed using Stata 18.0 and R 4.3.3 software packages. Two-tailed *p*-values were adopted for significance testing, with statistical significance defined as *p* < 0.05.

## Results

### Baseline characteristics of the study population

This cross-sectional analysis included 9,398 participants, of whom 464 were diagnosed with MetS. Compared with the low BRI group, the medium and high BRI groups exhibited significant demographic differences, including a higher proportion of females (*p* < 0.001), older mean age (*p* < 0.001), lower education level (*p* < 0.001), and a higher prevalence of ≥2 chronic diseases (*p* < 0.001), along with elevated BMI values, as detailed in Table 1.

**Table 1:** Baseline characteristics of the study population stratified by BRI in the cross-sectional analysis.

Variables	Total (*n* = 9,398)	Low BRI (*n* = 3,149)	Medium BRI (*n* = 3,108)	High BRI (*n* = 3,141)	*P*
Age (years)	59.00 (52.00, 65.00)	58.00 (52.00, 65.00)	58.00 (51.00, 65.00)	60.00 (53.00, 67.00)	*P* < 0.001
Gender (%)					*P* < 0.001
Males	4,404 (46.9)	2011 (63.9)	1,503 (48.4)	890 (28.3)	
Females	44,994 (53.1)	1,138 (36.1)	1,605 (51.6)	2,251 (71.7)	
Hukou status (%)					*P* < 0.001
Agriculture	7,755 (82.5)	2,734 (86.8)	2,525 (81.2)	2,496 (79.5)	
Non-agriculture	1,643 (17.5)	415 (13.2)	583 (18.8)	645 (20.5)	
Education (%)					*P* < 0.001
Illiterate	2,731 (29.1)	792 (25.1)	839 (27.0)	1,100 (35.0)	
Primary school or below	3,860 (41.1)	1,375 (43.7)	1,275 (41.0)	1,210 (38.5)	
Middle school	2,680 (28.5)	937 (29.8)	944 (30.4)	799 (25.4)	
High school or above	127 (1.3)	45 (1.4)	50 (1.6)	32 (1.1)	
Marital status (%)					0.006
Married	8,266 (87.9)	2,782 (88.3)	2,767 (89.0)	2,717 (86.5)	
Other	1,132 (12.1)	367 (11.7)	341 (11.0)	424 (13.5)	
Current smoking (%)					*P* < 0.001
Yes	3,737 (39.8)	1,693 (53.8)	1,229 (39.5)	815 (25.9)	
No	5,661 (60.2)	1,456 (46.2)	1879 (60.5)	2,326 (74.1)	
Current drinking (%)					*P* < 0.001
Yes	2,360 (25.1)	1,029 (32.7)	803 (25.8)	528 (16.8)	
No	7,038 (74.9)	2,120 (67.3)	2,305 (74.2)	2,613 (83.2)	
Sleep duration (hour)	6.00 (5.00, 8.00)	6.00 (5.00, 8.00)	6.00 (5.00, 8.00)	6.00 (5.00, 8.00)	0.601
Social activities (%)					0.002
Yes	4,747 (50.5)	1,519 (48.2)	1,573 (50.6)	1,655 (52.7)	
No	4,651 (49.5)	1,630 (51.8)	1,535 (49.4)	1,486 (47.3)	
Chronic disease (%)					*P* < 0.001
0	2,850 (30.3)	1,130 (35.9)	992 (31.9)	728 (23.2)	
1	2,854 (30.4)	965 (30.6)	949 (30.5)	940 (29.9)	
≥ 2	3,694 (39.3)	1,054 (33.5)	1,167 (37.6)	1,473 (46.9)	
BMI (kg/m^2^)	23.75 ± 10.76	20.60 ± 2.75	23.24 ± 2.68	27.39 ± 17.56	*P* < 0.001
SES level					*P* < 0.001
Q1	3,323 (35.4)	1,043 (33.1)	1,041 (33.5)	1,239 (35.5)	
Q2	2,829 (30.1)	977 (31.0)	933 (30.0)	919 (29.3)	
Q3	3,246 (34.5)	1,129 (35.9)	1,134 (36.5)	983 (31.3)	
MetS (%)					*P* < 0.001
Yes	464 (4.9)	20 (0.6)	112 (3.6)	332 (10.6)	
No	8,934 (95.1)	3,129 (99.4)	2,996 (96.4)	2,809 (89.4)	

Continuous variables with normal distribution are expressed as mean ± SD, while non-normally distributed variables are reported as median (IQR). Categorical variables are presented as numbers and percentages. BMI, Body Mass Index; MetS, Metabolic Syndrome; BRI, Body Roundness Index. The 33.3rd and 66.7th percentiles of the BRI were 3.47 and 4.72, respectively.

The 4-year prospective cohort study followed 5,934 participants, comprising 2,770 males (46.7%) and 3,164 females (53.3%), with a mean age of 58 years. Among these, 1,626 incident cases of MetS were identified, yielding an incidence rate of 27.4%. Prevalence and incidence of MetS in different BRI groups were detailed in the Supplementary Image 1. When stratified into BRI tertiles: low (*n* = 2040), medium (*n* = 2010), high (*n* = 1884), the medium and high BRI groups exhibited significantly higher proportions of female participants, lower education levels, greater chronic disease burden, and elevated BMI values compared to the low BRI group (all *p* < 0.001). Notably, MetS incidence increased progressively with higher BRI levels, with full statistical outcomes provided in Table 2.

**Table 2:** Baseline characteristics of the study population stratified by BRI in the longitudinal cohort analysis.

Variables	Total (*n* = 5,934)	Low BRI (*n* = 2040)	Medium BRI (*n* = 2010)	Higher BRI (*n* = 1884)	*P*
Age (years)	58.00 (52.00, 65.00)	58.00 (52.00, 65.00)	58.00 (52.00, 64.00)	59.00 (53.00, 66.00)	*P* < 0.001
Gender (%)					*P* < 0.001
Males	2,770 (46.7)	1,293 (63.4)	964 (48.0)	513 (27.2)	
Females	3,164 (53.3)	747 (36.6)	1,046 (52.0)	1,371 (72.8)	
Hukou status (%)					*P* < 0.001
Agriculture	5,064 (85.3)	1805 (88.5)	1,686 (83.9)	1,573 (83.5)	
Non-agriculture	870 (14.7)	235 (11.5)	324 (16.1)	311 (16.5)	
Education (%)					*P* < 0.001
Illiterate	1,699 (28.6)	497 (24.3)	556 (27.7)	646 (34.2)	
Primary school or below	2,501 (42.2)	903 (44.3)	846 (42.0)	752 (39.9)	
Middle school	1,667 (28.1)	613 (30.1)	580 (28.9)	474 (25.2)	
High school or above	67 (1.1)	27 (1.3)	28 (1.4)	12 (0.7)	
Marital status (%)					0.080
Married	5,281 (89.0)	1815 (89.0)	1811 (90.1)	1,655 (87.8)	
Other	653 (11.0)	225 (11.0)	199 (9.9)	229 (12.2)	
Current smoking (%)					*P* < 0.001
Yes	2,348 (39.6)	1,094 (53.6)	770 (38.3)	484 (25.7)	
No	3,586 (60.4)	946 (46.4)	1,240 (61.7)	1,400 (74.3)	
Current drinking (%)					*P* < 0.001
Yes	1,513 (25.5)	664 (32.5)	527 (26.2)	322 (17.1)	
No	4,421 (74.5)	1,376 (67.5)	1,483 (73.8)	1,562 (82.9)	
Sleep duration (hour)	6.00 (5.00, 8.00)	6.00 (5.00, 8.00)	6.00 (5.00, 8.00)	6.00 (5.00, 8.00)	0.669
Social activities (%)					0.051
Yes	2,966 (50.0)	979 (48.0)	1,010 (50.3)	977 (51.9)	
No	2,968 (50.0)	1,061 (52.0)	1,000 (49.7)	907 (48.1)	
Chronic disease (%)					*P* < 0.001
0	1924 (32.4)	754 (37.0)	676 (33.6)	494 (26.2)	
1	1842 (31.0)	608 (29.8)	615 (30.6)	619 (32.9)	
≥ 2	2,168 (36.6)	678 (33.2)	719 (35.8)	771 (40.9)	
BMI (kg/m^2^)	23.72 ± 12.06	20.66 ± 2.54	23.28 ± 2.69	27.50 ± 20.47	*P* < 0.001
SES level					0.001
Q1	2,126 (35.8)	683 (33.5)	698 (34.7)	745 (39.5)	
Q2	1839 (31.0)	649 (31.8)	617 (30.7)	573 (30.4)	
Q3	1969 (33.2)	708 (34.7)	695 (34.6)	566 (30.1)	
MetS (%)					*P* < 0.001
Yes	1,626 (27.4)	184 (9.0)	528 (26.3)	914 (48.5)	
No	4,308 (72.6)	1856 (91.0)	1,482 (73.7)	970 (51.5)	

Continuous variables with normal distribution are expressed as mean ± SD, while non-normally distributed variables are reported as median (IQR). Categorical variables are presented as numbers and percentages. BMI, Body Mass Index; MetS, Metabolic Syndrome; BRI, Body Roundness Index. The 33.3rd and 66.7th percentiles of the BRI were 3.44 and 4.65, respectively.

### Association between BRI and MetS

Table 3 presents the cross-sectional association between baseline BRI and MetS prevalence. In unadjusted models, BRI demonstrated a positive association with MetS risk (OR = 5.85, 95% CI: 3.62–9.44), with participants in the high BRI tertile exhibiting substantially elevated risk (OR = 18.49, 95% CI: 11.74–29.12; *p* < 0.001). Following full adjustment for covariates, including demographic factors, lifestyle behaviors, and health-related variables, the association remained significant for both the medium BRI tertile (OR = 4.99, 95% CI: 3.07–8.11) and high BRI tertile (OR = 13.66, 95% CI: 8.57–21.79) compared to the low BRI group (all p < 0.001).

**Table 3:** Cross-sectional association between baseline BRI and MetS.^a^

MetS	OR (95% CI)		
	Low BRI (*n* = 3,149)	Medium BRI (*n* = 3,108)	High BRI (*n* = 3,141)
Model 1	1.0 (reference)	5.85 (3.62, 9.44)^***^	18.49 (11.74, 29.12) ^***^
Model 2	1.0 (reference)	5.47 (3.39, 8.85) ^***^	17.29 (10.91, 27.39) ^***^
Model 3	1.0 (reference)	4.99 (3.07, 8.11) ^***^	13.66 (8.57, 21.79) ^***^

^a^Logistic regression. OR, odd ratio; CI, confidence interval; MetS, Metabolic Syndrome; BRI, Body Roundness Index. Model 1 = adjust for none; Model 2 = age + gender + Hukou status + marital status + education; Model 3 = Model 2 + current smoking + current drinking + sleep duration + social activities + chronic disease + BMI + SES. ^***^p < 0.001. The 33.3rd and 66.7th percentiles of the BRI were 3.47 and 4.72, respectively.

The longitudinal analysis included 5,934 participants over a 4-year follow-up period, during which 1,626 incident cases of MetS were identified. Table 4 summarizes the longitudinal relationship between baseline BRI and MetS incidence. Longitudinal findings aligned closely with cross-sectional observations. After adjusting for potential confounders, participants in the medium BRI tertile exhibited a 2.71-fold increased risk of MetS development compared to the low BRI group (HR = 2.71, 95% CI: 2.29–3.21), while those in the high BRI tertile demonstrated a 4.64-fold elevated risk (HR = 4.64, 95% CI: 3.94–5.47; both *p* < 0.001).

**Table 4:** Longitudinal association between BRI and MetS during follow-up.^b^

MetS	HR (95% CI)		
	Low BRI	Medium BRI	High BRI
Model 1	1.0 (reference)	2.91 (2.46, 3.44) ^***^	5.38 (4.59, 6.30) ^***^
Model 2	1.0 (reference)	2.72 (2.29, 3.22) ^***^	4.75 (4.04, 5.59) ^***^
Model 3	1.0 (reference)	2.71 (2.29, 3.21) ^***^	4.64 (3.94, 5.47) ^***^

^b^Cox Proportional Hazards Regression. HR, Hazard Ratio; CI, confidence interval; MetS, Metabolic Syndrome; BRI, Body Roundness Index. Model 1 = adjust for none; Model 2 = age + gender + Hukou status + marital status + education; Model 3 = Model 2 + current smoking + current drinking + sleep duration + social activities + chronic disease + BMI + SES. ^***^P<0.001. The 33.3rd and 66.7th percentiles of the BRI were 3.44 and 4.65, respectively.

### Dose–response relationship between BRI and MetS incidence

**Figure 2:**
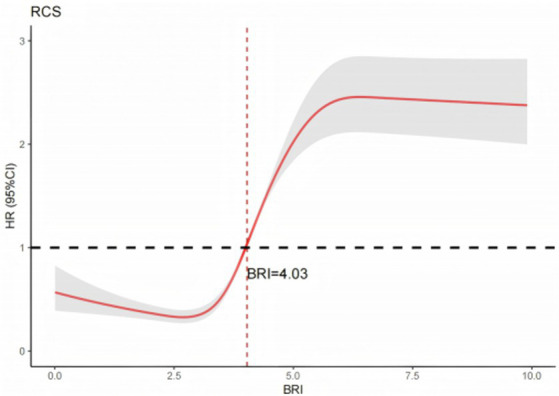
Dose-response relationship between BRI and MetS.

Additionally, this study utilized restricted cubic spline (RCS) analysis to evaluate the dose–response relationship between BRI and MetS incidence. Consistent with findings from logistic and Cox regression models, elevated BRI levels were robustly associated with an increased risk of MetS. The RCS curve, adjusted for covariates in Model 3, revealed a statistically significant nonlinear dose–response relationship (P for nonlinearity < 0.001). Inflection point analysis identified a critical risk threshold at BRI = 4.03: below this threshold, incremental increases in BRI demonstrated a protective or neutral effect, whereas above this threshold, increments in BRI were associated with a marked exponential increase in MetS incidence. Full analytical results are presented in Figure 2.

### Subgroup analysis

To further investigate the association between BRI and MetS, stratified subgroup analyses were conducted. Results demonstrated significant associations between elevated BRI levels and MetS risk in most subgroups. Compared to the low BRI group, participants aged ≥60 years in the medium and high BRI tertiles had significantly higher risks (medium BRI: HR = 3.28, 95% CI: 2.47–4.36; high BRI: HR = 7.60, 95% CI: 5.84–9.88). Notably, within the higher BRI tertile, males exhibited a substantially higher MetS risk (HR = 9.02, 95% CI: 6.93–11.73) compared to females (HR = 4.47, 95% CI: 3.65–5.47). Significant interaction effects were observed between BRI and MetS across gender, Hukou status, smoking status, drinking status, BMI, and chronic disease (all *p* < 0.05). However, no significant association was observed among individuals with tertiary education or higher (*p* > 0.05). Full results of the stratified analyses are presented in Table 5.

**Table 5:** Stratified subgroup analysis of the association between BRI and MetS.

Characteristics	HR (95% CI)			P for interaction
	Low BRI	Medium BRI	High BRI	
Age (years)				0.49
< 60	1.0 (reference)	3.15 (2.56, 3.88) ^***^	6.81 (5.58, 8.32) ^***^	
≥ 60	1.0 (reference)	3.28 (2.47, 4.36) ^***^	7.60 (5.84, 9.88) ^***^	
Gender				*p* < 0.001
Male	1.0 (reference)	3.93 (3.01, 5.12) ^***^	9.02 (6.93, 11.73) ^***^	
Female	1.0 (reference)	2.36 (1.90, 2.93) ^***^	4.47 (3.65, 5.47) ^***^	
Hukou status				0.004
Agriculture	1.0 (reference)	3.27 (2.72, 3.94) ^***^	7.61 (6.39, 9.07) ^***^	
Non-agriculture	1.0 (reference)	2.67 (1.80, 3.94) ^***^	4.10 (2.81, 5.99) ^***^	
Marital status				0.757
Married	1.0 (reference)	3.18 (2.67, 3.80) ^***^	7.03 (5.95, 8.32) ^***^	
Other	1.0 (reference)	3.50 (2.07, 5.90) ^***^	6.45 (3.95, 10.54) ^***^	
Education				0.064
Illiterate	1.0 (reference)	2.55 (1.84, 3.53) ^***^	5.79 (4.29, 7.81) ^***^	
Primary school or below	1.0 (reference)	3.53 (2.69, 4.62) ^***^	8.22 (6.36, 10.62) ^***^	
Middle school	1.0 (reference)	3.78 (2.80, 5.11) ^***^	7.37 (5.51, 9.86) ^***^	
High school or above	1.0 (reference)	1.10 (0.42, 2.85)	2.38 (0.86, 6.59)	
Current smoking				*p* < 0.001
Yes	1.0 (reference)	4.03 (3.05, 5.34) ^***^	9.41 (7.17, 12.36) ^***^	
No	1.0 (reference)	2.54 (2.06, 3.14) ^***^	5.17 (4.25, 6.30) ^***^	
Current drinking				0.023
Yes	1.0 (reference)	4.63 (3.23, 6.62) ^***^	9.52 (6.68, 13.58) ^***^	
No	1.0 (reference)	2.81 (2.32, 3.39) ^***^	6.06 (5.07, 7.24) ^***^	
BMI (kg/m^2^)				*P* < 0.001
< 24	1.0 (reference)	3.02 (2.47, 3.68) ^***^	5.51 (4.35, 6.98) ^***^	
≥ 24	1.0 (reference)	1.23 (0.86, 1.75)	2.05 (1.46, 2.87) ^***^	
Chronic disease				0.006
0	1.0 (reference)	3.01 (2.27, 3.98) ^***^	6.62 (5.05, 8.66) ^***^	
1	1.0 (reference)	4.87 (3.45, 6.87) ^***^	10.41 (7.49, 14.47) ^***^	
≥ 2	1.0 (reference)	2.51 (1.92, 3.29) ^***^	5.40 (4.21, 6.92) ^***^	
SES				0.511
Q1	1.0 (reference)	2.61 (1.95, 3.49) ^***^	6.79 (5.19, 8.87) ^***^	
Q2	1.0 (reference)	4.14 (3.04, 5.66) ^***^	8.00 (5.93, 10.79) ^***^	
Q3	1.0 (reference)	3.13 (2.38, 4.12) ^***^	6.35 (4.88, 8.26) ^***^	

HR, Hazard Ratio; CI, confidence interval; BMI, Body Mass Index; MetS, Metabolic Syndrome; BRI, Body Roundness Index. ***P < 0.001.

### Sensitivity analysis

To evaluate the robustness of the findings, MetS was redefined using objective laboratory and anthropometric data (excluding self-reported diagnoses), aligning with the original ATP III criteria (38). Using a sample selection protocol parallel to the primary analysis, 4,141 participants with complete follow-up data were included, among whom 13.4% met the revised MetS criteria. Cox regression analyses produced consistent results, confirming that objectively defined MetS remained similarly associated with BRI (Table 6). Moreover, after excluding participants in the top and bottom 15% of baseline BRI values, the associations persisted with minimal attenuation. Following full adjustment for covariates, participants in the medium and high BRI tertiles had a 2.34-fold (HR = 2.34, 95% CI: 1.94–2.81) and 3.36-fold (HR = 3.36, 95% CI: 2.77–4.08) increased risk of MetS, respectively, compared to the low BRI group (both *p* < 0.001; Table 6).

**Table 6:** Sensitivity analysis of BRI and MetS during the follow-up period.^b^

MetS	HR (95% CI)		
	Low BRI	Medium BRI	Higher BRI
Definition of MetS using only blood test and physical examination data^c^			
Model 1	Reference	2.44 (1.97, 3.02) ^***^	4.10 (3.30, 5.09) ^***^
Model 2	Reference	2.28 (1.84, 2.83) ^***^	3.57 (2.85, 4.47) ^***^
Model 3	Reference	2.29 (1.85, 2.85) ^***^	3.51 (2.79, 4.41) ^***^
Exclusion of participants with an extreme baseline BRI of 15 per cent^d^			
Model 1	Reference	2.81 (2.35, 3.36) ^***^	4.86 (4.10, 5.76) ^***^
Model 2	Reference	2.65 (2.21, 3.17) ^***^	4.39 (3.69, 5.22) ^***^
Model 3	Reference	2.34 (1.94, 2.81) ^***^	3.36 (2.77, 4.08) ^***^

^b^Cox Proportional Hazards Regression. ^c^The 33.3rd and 66.7th percentiles of the BRI were 3.17 and 4.10, respectively. ^d^The 33.3rd and 66.7th percentiles of the BRI were 3.54 and 4.56, respectively. HR, Hazard Ratio; CI, confidence interval; MetS, Metabolic Syndrome; BRI, Body Roundness Index. Model 1 = adjust for none; Model 2 = age + gender + Hukou status + marital status + education; Model 3 = Model 2 + current smoking + current drinking + sleep duration + social activities + chronic disease + BMI+ SES. ***P < 0.001.

## Discussion

The findings of this study demonstrate a significant positive association between BRI and MetS risk. Both cross-sectional and longitudinal analyses revealed that participants in the medium and high BRI exhibited had substantially elevated risks of MetS compared to those in the low BRI group. This association remained significant after adjusting for covariates (*p* < 0.001). Restricted cubic spline analysis further revealed a nonlinear dose–response relationship between BRI and MetS risk (P for nonlinearity *p* < 0.001), with a critical threshold identified at BRI = 4.03. Notably, the association between BRI and MetS was more pronounced in specific subgroups, including males, individuals aged ≥ 60 years, those with a history of smoking or alcohol use, and participants with chronic diseases (*p* < 0.001).

Previous studies have primarily compared BRI with other anthropometric indices, such as WC and BMI, in predicting MetS risk factors (39–41). For example, a cross-sectional study of older Turkish adults demonstrated that BRI had superior predictive accuracy for MetS compared to conventional anthropometric measures, showing the highest area under the curve (AUC) among evaluated indices: 0.678 (95% CI: 0.591–0.764) for males and 0.645 (95% CI: 0.568–0.723) for females (42). Similarly, Stefanescu A et al. evaluated the predictive performance of BRI, BMI, WC and other indices for MetS in 1,815 Peruvian adults and identified BRI as a robust predictor. Each unit increase in BRI was associated with a 2.43-fold rise in odds in males (OR = 2.43; 95% CI: 1.95–3.02) and a 1.89-fold increase in females (OR = 1.89; 95% CI: 1.68–2.12) (43). Another study found that among novel anthropometric indices, participants in the highest BRI quartile had the greatest risk of MetS and its components, with the fourth quartile showing the highest odds ratio (OR = 66.03; 95% CI: 18.01–242.1) (44). Additionally, Liu B. et al. reported a strong independent association between BRI and MetS odds in both genders after adjusting for age, diabetes history, and BMI (*p* < 0.001). Compared to the lowest tertile of BRI, higher tertiles were associated with significantly increased odds of MetS (males: second tertile OR = 5.053, third tertile OR = 7.195; females: second tertile OR = 4.616, third tertile OR = 3.772) (30). Our findings corroborate this evidence, further supporting a significant positive association between BRI and MetS risk. The observed associations suggest that systematic incorporation of BRI measurements may warrant consideration in preventive geriatric healthcare frameworks, particularly for enhancing metabolic risk stratification in community health screenings and primary care settings serving aging populations.

Emerging evidence underscores the critical role of modifiable lifestyle factors and environmental exposures in modulating MetS risk. A meta-analysis demonstrated that impaired sleep quality significantly elevates MetS incidence (OR = 1.37, 95% CI = 1.15–1.64), though with notable heterogeneity (I^2^ = 62.4%, *p* < 0.1) (45). Conversely, physical activity exhibits dose-dependent protective effects: the results show that a 36% risk reduction in the highest versus lowest activity quartile (OR = 0.64, 95% CI = 0.55–0.73) (46), while a longitudinal analyses identified an 8% attenuation in MetS risk per 10 MET-hour/week increment in leisure-time physical activity (*β* = −0.08, *p* < 0.01) (47). Dietary interventions further modulate cardiometabolic profiles, with randomized trials showing Mediterranean diets significantly lowering total cholesterol (MD = -7.97 mg/dL, 95% CI = -14.82 to −1.11) and systolic blood pressure (MD = -2.04 mmHg, 95% CI = -3.68 to −0.39) compared to low-fat regimens (48). Environmental toxicology studies highlight synergistic risks, as chronic exposure to traffic-related nitrogen oxides (>50 parts per billion) and noise (>65 decibels) in Mexican American cohorts increased hypoalphalipoproteinemia (HR = 1.22, 95% CI = 1.05–1.41) and MetS incidence (HR = 1.18, 95% CI = 1.02–1.36) (49). Importantly, multimodal interventions integrating supervised exercise (150 min/week) and dietary modification achieved clinically meaningful reductions in fasting glucose and systolic blood pressure over 6 months, demonstrating actionable pathways for MetS management (50).

Abdominal obesity is characterized by adipocyte hyperplasia and hypertrophy. These morphological changes can lead to adipose tissue dysfunction, including dysregulated secretion of both anti-inflammatory and pro-inflammatory cytokines and impaired free fatty acid metabolism, collectively increasing the risk of metabolic syndrome (51–53). Compared to imaging-based assessments of visceral fat, WC measured via tape provides a more accessible and practical method for evaluating abdominal adiposity. However, a critical limitation of WC is its inability to account for height variation, which may result in underestimating abdominal obesity in taller individuals or overestimating it in shorter populations (54). To address this, researchers developed the BRI, a novel anthropometric index calculated by normalizing WC to height. BRI demonstrates superior accuracy to WC in predicting both total body fat percentage and visceral adipose tissue accumulation (26).

The potential mechanisms linking BRI and MetS risk may include the following pathways. First, BRI is associated with hypertension, dyslipidemia, and diabetes mellitus—established MetS risk factors that may mutually contribute to its development (55–58). Second, obesity-induced chronic inflammation is intricately linked to metabolic syndrome, primarily mediated by adipose tissue dysfunction and imbalanced adipokine secretion (51). In obesity, pro-inflammatory adipokines such as tumor necrosis factor (TNF) and interleukin-6 (IL-6) are upregulated in adipose tissue, triggering chronic low-grade inflammation that impairs systemic metabolic regulation (59, 60). These inflammatory mediators disrupt insulin signaling pathways, leading to insulin resistance. Additionally, immune cell infiltration (particularly macrophages) into adipose tissue and their phenotypic switching are closely associated with both chronic inflammation and the pathogenesis of insulin resistance (61, 62). One study demonstrates reduced soluble epoxide hydrolase (sEH) activity and implicates fatty acid diols in white adipose tissue (WAT) and liver during metabolic syndrome, suggesting novel mechanistic pathways (63). Thirdly, dysbiosis disrupts bile acid homeostasis and Farnesoid X receptor activation, impairing metabolic regulation and promoting dyslipidemia and chronic inflammation—key hallmarks of MetS progression to diabetes (8, 64). MetS originates from energy imbalance, genetic/epigenetic, and lifestyle factors, mediated through free fatty acid-induced insulin resistance, IL-6/TNF-*α*-mediated inflammation, and fetuin-A-driven mitochondrial reactive oxygen species dysregulation. Combination therapies including statins and probiotics, along with dietary interventions such as the Mediterranean diet and time-restricted eating, have been shown to mitigate these metabolic abnormalities (65).

This investigation demonstrates multiple methodological strengths, most notably as the first prospective cohort study to investigate the association between BRI and MetS in middle-aged and older Chinese adults. The use of nationally representative CHARLS data enhances the generalizability of the findings. Furthermore, the research design extends beyond cross-sectional analyses to incorporate longitudinal assessments, providing comprehensive insights into the temporal relationships between BRI fluctuations and MetS risk progression.

Several limitations should be acknowledged. First, the exclusive focus of the CHARLS dataset on a Chinese population limits the generalizability of these findings to other ethnic or cultural groups. Second, the temporal scope of the study—particularly the four-year follow-up period from 2011 to 2015—might be perceived as insufficient for capturing long-term metabolic trajectories. Nevertheless, the significant associations identified within this timeframe highlight the clinical relevance of BRI as an early biomarker. Third, while this study employed the NCEP ATP III diagnostic criteria for MetS, potential discrepancies may exist between these criteria and other established definitions. Fourth, although adjustments were implemented for *a priori*-identified confounding variables, residual confounding persists due to unmeasured factors such as physical activity patterns and dietary intake. Finally, reliance on self-reported data introduces potential recall bias, which could affect the accuracy of parameter estimates. Given these limitations, prospective cohort studies incorporating longitudinal designs are warranted to elucidate the precise mechanisms through which BRI contributes to incident MetS.

## Conclusion

In conclusion, moderate and high BRI groups showed a significant association with an increased risk of MetS among middle-aged and older Chinese adults. These findings reinforce the evidence that elevated BRI adversely affects health in this population. Middle-aged and older adults with moderate to high BRI should undergo regular MetS screening and receive preventive health education in clinical settings.

## Data Availability

The original contributions presented in the study are included in the article/Supplementary material, further inquiries can be directed to the corresponding author.

## Glossary

BRI: Body Roundness Index
MetS: Metabolic Syndrome
CHARLS: China Health and Retirement Longitudinal Study
OR: Odds Ratio
HR: Hazard Ratio
CI: Confidence interval
TG: Triglycerides
HDL-C: High-density Lipoprotein Cholesterol
BMI: Body Mass Index
WC: Waist Circumference
WHR: Waist-to-Hip Ratio
SBP: Systolic Blood Pressure
DBP: Diastolic Blood Pressure
FBG: Fasting Blood Glucose
M: Mean
SD: Standard Deviation
ANOVA: Analysis of Variance
RCS: Restricted Cubic Splines
AUC: Area under the Curve
TNF: Tumor Necrosis Factor
IL-6: Interleukin-6
