# Supplementary figures and images for: Association between body roundness index and metabolic syndrome in middle-aged and older adults: a prospective cohort study in China
Source: Front Public Health. 2025 Jul 9;13:1604593. doi: 10.3389/fpubh.2025.1604593 (PMC12283791; doi:10.3389/fpubh.2025.1604593)

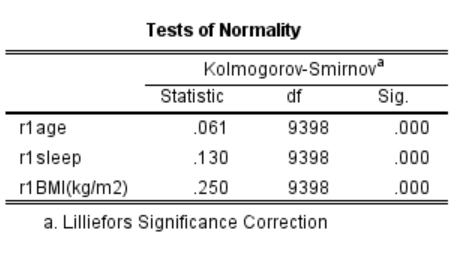


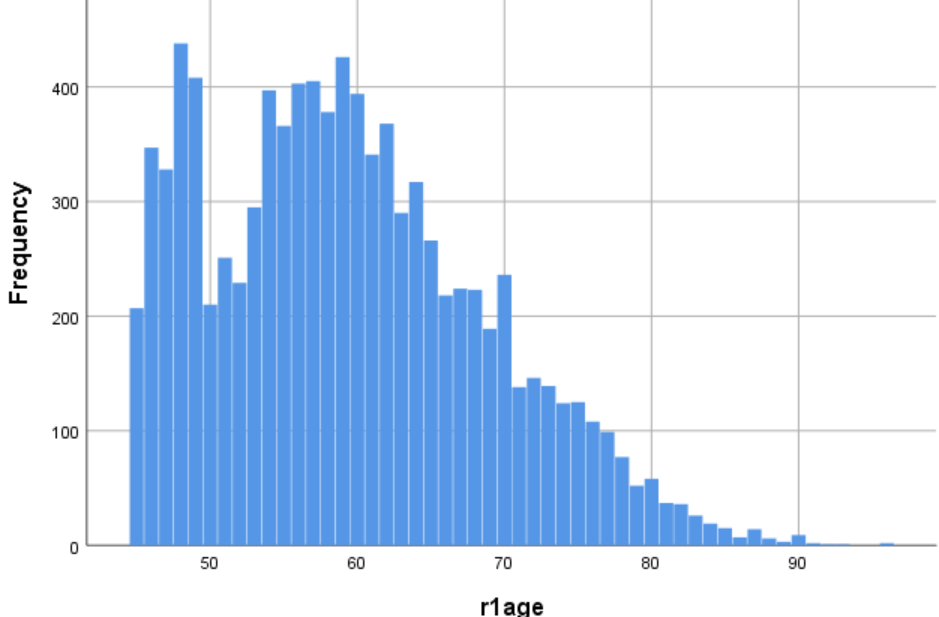


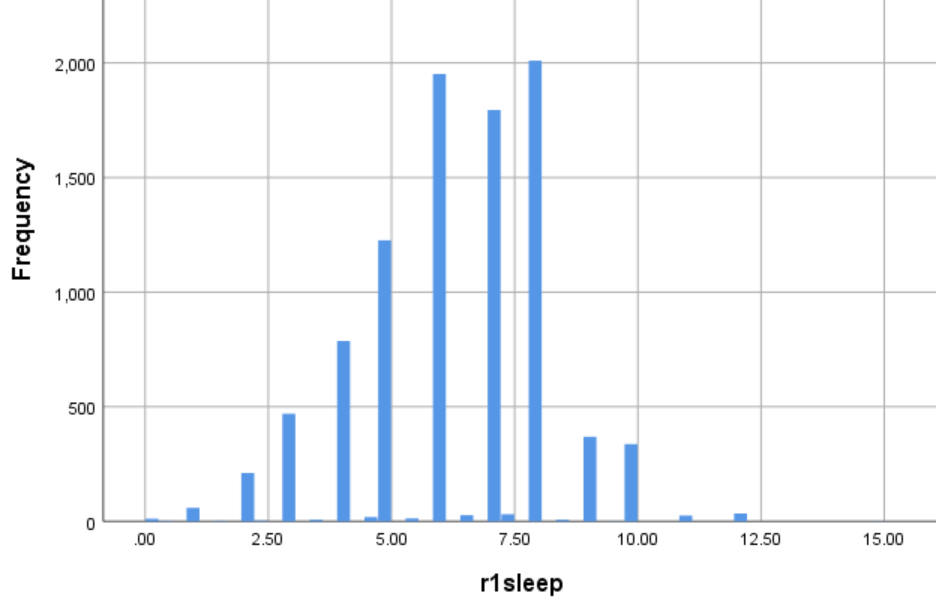


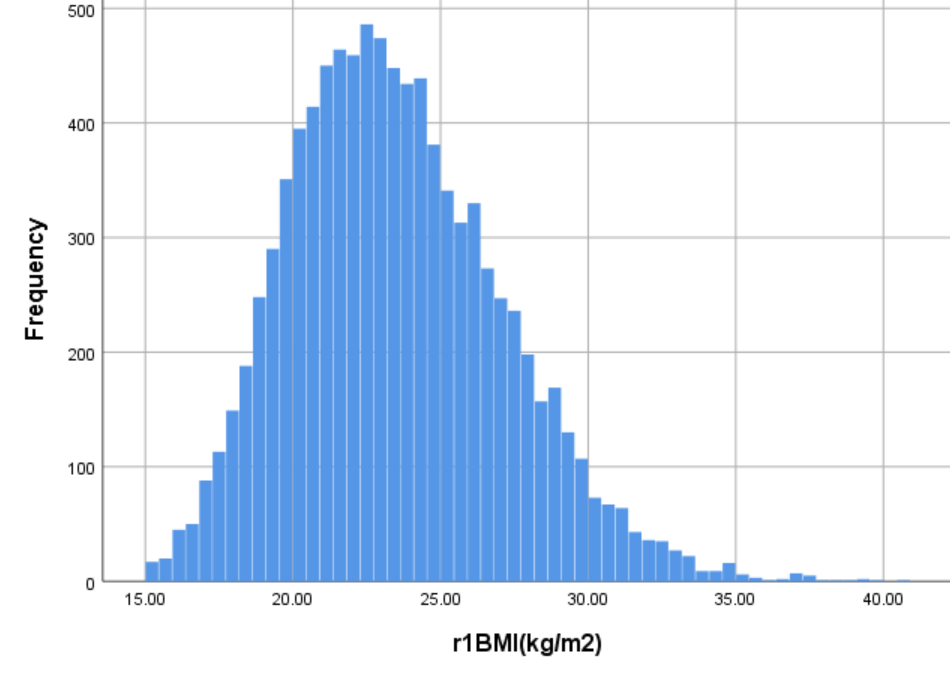

Supplement: Supplementary file 1 [file Table_1.docx]

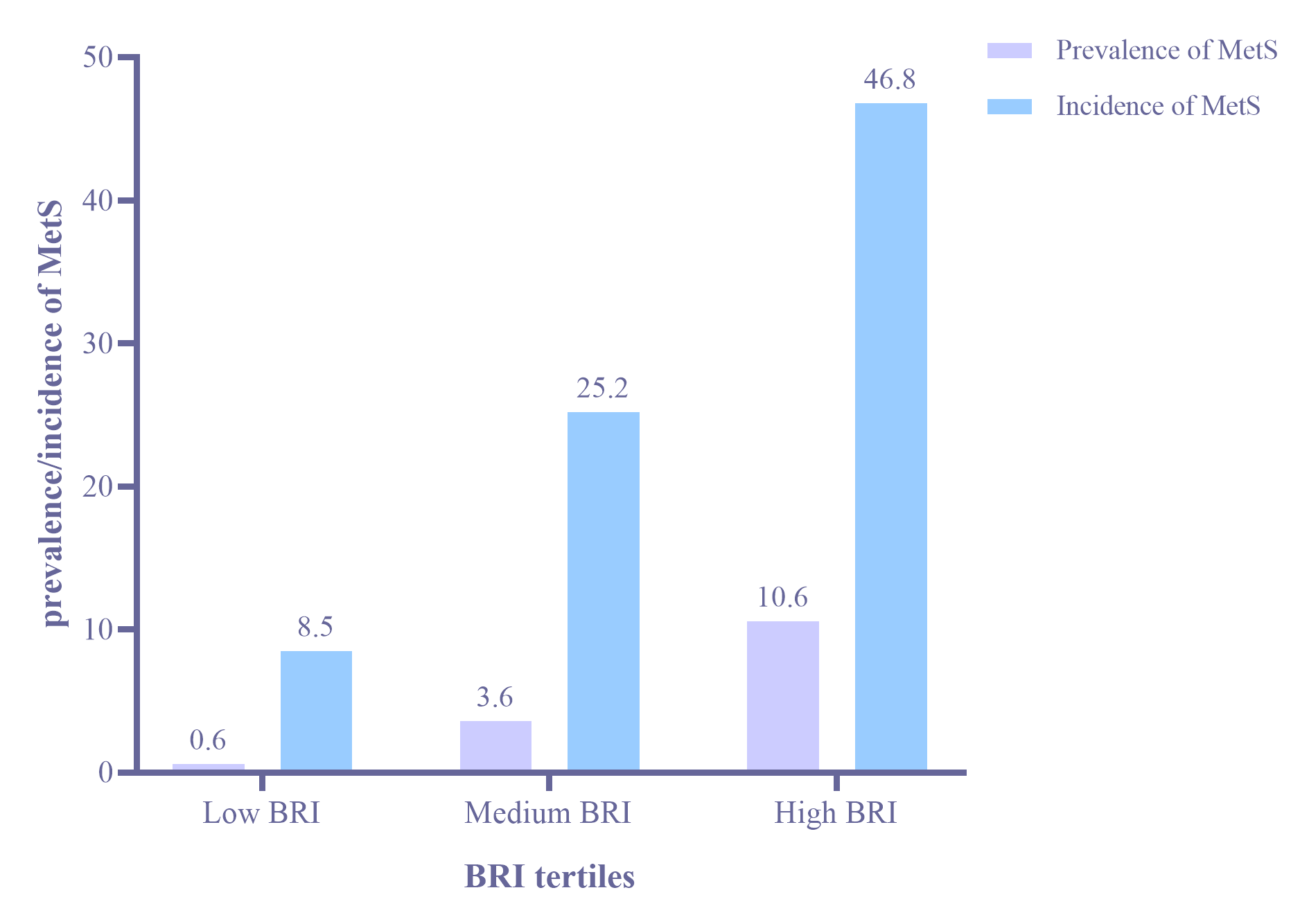

Supplement: Supplementary file 2 [file Image_1.tif]
